# Supplementary material for: Trans-Ethnic Polygenic Analysis Supports Genetic Overlaps of Lumbar Disc Degeneration With Height, Body Mass Index, and Bone Mineral Density
Source: Front Genet. 2018 Aug 3;9:267. doi: 10.3389/fgene.2018.00267 (PMC6088183; doi:10.3389/fgene.2018.00267)
Supplement: Supplementary file 1 [file Table_1.PDF]

Table S1 Basic demographic information and phenotype summary of the HKDD cohort.

(A) Demographic information

	All	Male	Female
Sample used in analysis	2,054	831	1223
Age at MRI	43.69±8.16	43.37±8.45	43.91±7.94
Height (m)	1.63±0.09	1.70±0.06	1.58±0.06
Weight (kg)	61.72±11.34	69.60±9.96	56.36±8.80
Body mass index (kg/m^2)	23.22±3.46	24.02±3.07	22.68±3.60
History of lumbar injury	33.79%	41.40%	28.62%

(B) Lumbar disc degeneration

Level		All	Male	Female
L1/L2	Grade 1	5.01%	5.90%	4.42%
	Grade 2	2.19%	2.29%	2.13%
	Grade 3	2.92%	4.21%	2.04%
L2/L3	Grade 1	9.30%	9.51%	9.16%
	Grade 2	5.60%	5.66%	5.56%
	Grade 3	3.41%	3.73%	3.19%
L3/L4	Grade 1	17.43%	20.34%	15.45%
	Grade 2	10.56%	10.23%	10.79%
	Grade 3	4.58%	4.69%	4.50%
L4/L5	Grade 1	19.77%	20.70%	19.13%
	Grade 2	23.81%	22.26%	24.86%
	Grade 3	11.78%	14.56%	9.89%
L5/S1	Grade 1	15.19%	16.97%	13.98%
	Grade 2	28.68%	25.87%	30.58%
	Grade 3	14.22%	17.57%	11.94%
Disc degeneration score		3.19±2.68	3.40±2.67	3.05±2.67

Disc Degeneration Grade 1: slight decrease in signal intensity in nucleus pulposus; Grade 2: generalized hypointense nucleus with normal disc height; Grade 3: generalized hypointense nucleus with disc space narrowing.

(C) Lumbar disc displacement

Level		All	Male	Female
L1/L2	Grade 1	1.02%	1.68%	0.57%
	Grade 2	0.05%	0.00%	0.08%
L2/L3	Grade 1	3.12%	3.73%	2.70%
	Grade 2	0.19%	0.12%	0.25%
L3/L4	Grade 1	7.50%	7.82%	7.28%
	Grade 2	0.10%	0.12%	0.08%
L4/L5	Grade 1	25.85%	27.08%	25.02%
	Grade 2	1.61%	2.05%	1.31%
L5/S1	Grade 1	32.91%	35.50%	31.15%
	Grade 2	1.85%	2.53%	1.39%
Disc displacement score		0.78±0.94	0.85±0.98	0.73±0.91

Disc Displacement Grade 1 (bulging/protrusion): disc has protruded posteriorly beyond a line connecting the posterior margins of the adjacent vertebral bodies; Grade 2 (extrusion): displacement of the nucleus beyond the confines of the annulus fibrosus.

The lumbar spine has 5 intervertebral segments, termed lumbar segment 1 through 5 (L1~L5). S1 stands for the first segment of sacral that is intermediately below the lumbar spine.
