# Supplementary material for: Trans-Ethnic Polygenic Analysis Supports Genetic Overlaps of Lumbar Disc Degeneration With Height, Body Mass Index, and Bone Mineral Density
Source: Front Genet. 2018 Aug 3;9:267. doi: 10.3389/fgene.2018.00267 (PMC6088183; doi:10.3389/fgene.2018.00267)
Supplement: Supplementary file 2 [file Table_2.PDF]

Table S2 Pairwise Pearson correlations between phenotypes in the HKDD cohort.

(a) Correlations between original phenotypes

	Height	Weight	BMI	Lumbar spine injury	Disc degeneration score
Weight	0.627 ***				
BMI	-0.004	0.757 ***			
Lumbar spine injury	0.126 ***	0.134 ***	0.081 ***		
Disc degeneration score	0.0742 ***	0.157 ***	0.148 ***	0.187 ***	
Disc displacement score	0.1045 ***	0.156 ***	0.115 ***	0.202 ***	0.569 ***

Significance codes: '***' p<0.001; '**' 0.001≤p<0.01; '*' 0.01≤p<0.05; '·' 0.05≤p<0.1

(b) Partial correlations between phenotypes after adjusting for age and gender.

	Height	Weight	BMI	Lumbar spine injury	Disc degeneration score
Weight	0.421 ***				
BMI	-0.092 ***	0.855 ***			
Lumbar spine injury	0.047 *	0.080 ***	0.066 **		
Disc degeneration score	0.115 ***	0.151 ***	0.107 ***	0.031	
Disc displacement score	0.083 ***	0.140 ***	0.110 ***	0.010	0.573 ***

Significance codes: '***' p<0.001; '**' 0.001≤p<0.01; '*' 0.01≤p<0.05; '·' 0.05≤p<0.1
