# Supplementary material for: Trans-Ethnic Polygenic Analysis Supports Genetic Overlaps of Lumbar Disc Degeneration With Height, Body Mass Index, and Bone Mineral Density
Source: Front Genet. 2018 Aug 3;9:267. doi: 10.3389/fgene.2018.00267 (PMC6088183; doi:10.3389/fgene.2018.00267)
Supplement: Supplementary file 3 [file Table_3.PDF]

Table S3 Multiple linear regression analysis of disc degeneration and disc displacement scores. The best fitting model is indicated by the square bracket.

(a) Disc degeneration score

	Model A	Model B	Model C	[Model D]	Model E	Model F
Age	0.110 ***<br>(0.005)	0.115 ***<br>(0.005)	0.101 ***<br>(0.005)	0.106 ***<br>(0.005)	0.107 ***<br>(0.002)	0.107 ***<br>(0.005)
Sex (Male:1, Female:2)	-0.217 *<br>(0.093)	0.258 ·<br>(0.133)	-0.086<br>(0.097)	0.496 ***<br>(0.137)	0.314 **<br>(0.116)	0.509 ***<br>(0.137)
Lumbar spine injury	1.026 ***<br>(0.098)	1.046 ***<br>(0.099)	1.017 ***<br>(0.099)	0.992 ***<br>(0.099)	0.999 ***<br>(0.099)	0.990 ***<br>(0.099)
Height		3.777 ***<br>(0.759)		4.563***<br>(0.762)		6.849 **<br>(2.179)
BMI			0.082 ***<br>(0.014)	0.095***<br>(0.014)		0.166 *<br>(0.065)
Weight					0.039***<br>(0.005)	-0.028<br>(0.025)
Adjusted R-squared	0.193	0.202	0.205	0.215	0.212	0.215

Significance codes: ‘***’ $p<0.001$; ‘**’ $0.001\leq p<0.01$; ‘*’ $0.01\leq p<0.05$; ‘·’ $0.05\leq p<0.1$

(b) Disc displacement score

	Model A	Model B	Model C	[Model D]	Model E	Model F
Age	0.017 ***<br>(0.002)	0.018 ***<br>(0.002)	0.013 ***<br>(0.002)	0.015 ***<br>(0.002)	0.014 ***<br>(0.002)	0.015 ***<br>(0.002)
Sex (Male:1, Female:2)	-0.059 ·<br>(0.034)	0.094 ·<br>(0.049)	-0.013<br>(0.035)	0.184 ***<br>(0.050)	0.143<br>(0.042)	0.178 ***<br>(0.050)
Lumbar spine injury	0.391 ***<br>(0.036)	0.391 ***<br>(0.036)	0.378 ***<br>(0.036)	0.370 ***<br>(0.036)	0.371 ***<br>(0.036)	0.371 ***<br>(0.036)
Height		1.245 ***<br>(0.277)		1.543 ***<br>(0.278)		0.604<br>(0.795)
BMI			0.032***<br>(0.005)	0.036***<br>(0.005)		0.007<br>(0.024)
Weight					0.015***<br>(0.002)	0.011<br>(0.009)
Adjusted R-squared	0.073	0.081	0.086	0.096	0.096	0.096

Significance codes: ‘***’ p<0.001; ‘**’ 0.001≤p<0.01; ‘*’ 0.01≤p<0.05; ‘·’ 0.05≤p<0.1
