# Supplementary material for: Trans-Ethnic Polygenic Analysis Supports Genetic Overlaps of Lumbar Disc Degeneration With Height, Body Mass Index, and Bone Mineral Density
Source: Front Genet. 2018 Aug 3;9:267. doi: 10.3389/fgene.2018.00267 (PMC6088183; doi:10.3389/fgene.2018.00267)
Supplement: Supplementary file 4 [file Table_4.PDF]

Table S4 Parameters of best fit genetic models. When fitting the theoretical model to observed PGS results, we fixed SNP heritability to several plausible values then estimated the fraction of null markers and genetic covariance between discovery and testing samples.

Phenotype	Discovery GWAS sample size N_1	Number of independent SNPs M	Inflation factor[§]	LD-score regression intercept	SNP heritability h_1^2	Fraction of null SNPs $\hat{\pi}_0$	Genetic covariance $\hat{\sigma}_{12}$
Height	252,220	77,219	1.20	2.011	0.42	0.834	0.181
					0.35	0.833	0.172
					0.4	0.834	0.178
					0.45	0.835	0.185
BMI	233,719	78,306	1.37	1.295	0.22	0.846	0.099
					0.15	0.876	0.091
					0.20	0.854	0.097
					0.25	0.834	0.102
LS-BMD	29,300	76,829	1.127	0.966	0.37	0.995	0.167
					0.25	0.994	0.149
					0.30	0.995	0.156
					0.35	0.995	0.163
FN-BMD	31,300	78,010	1.112	0.956	0.34	0.994	0.175
					0.25	0.994	0.160
					0.30	0.994	0.168
					0.35	0.994	0.176

[§] Inflation factor: We amplified z-scores of GWAS summary data by this constant so that LDSC regression estimate of SNP heritability was consistent with the expected value (first line of each trait). The observed p-value thresholds were then corrected according to Appendix 2.
