# Supplementary material for: Trans-Ethnic Polygenic Analysis Supports Genetic Overlaps of Lumbar Disc Degeneration With Height, Body Mass Index, and Bone Mineral Density
Source: Front Genet. 2018 Aug 3;9:267. doi: 10.3389/fgene.2018.00267 (PMC6088183; doi:10.3389/fgene.2018.00267)
Supplement: Supplementary file 5 [file Table_5.PDF]

Table S5 Association of the BMI polygenic score with disc herniation and degeneration scores after adjusting for different sets of covariates.

Target phenotype	Covariates	sgn(β)	R^2	*p*-value
Disc displacement score	Age, Sex, Lumbar Injury	+	**0.287%**	**1.45E-02**
	Age, Sex	+	**0.233%**	**2.63E-02**
	Age, Sex, Lumbar Injury, Height	+	**0.241%**	**2.60E-02**
	Age, Sex, Lumbar Injury, BMI	+	0.076%	2.11E-01
	Age, Sex, Lumbar Injury, Height, BMI	+	0.069%	2.34E-01
	Age, Sex, Spine Injury, Weight	+	0.066%	2.45E-01
Disc degeneration score	Age, Sex, Lumbar Injury	+	**0.311%**	**1.08E-02**
	Age, Sex	+	**0.217%**	**3.16E-02**
	Age, Sex, Lumbar Injury, Height	+	**0.297%**	**1.35E-02**
	Age, Sex, Lumbar Injury, BMI	+	0.133%	9.82E-02
	Age, Sex, Lumbar Injury, Height, BMI	+	0.123%	1.12E-01
	Age, Sex, Lumbar Injury, Weight	+	0.105%	1.42E-01

The BMI PGS was created at the optimal p-value threshold ($P \leq 0.0123$) in the HKDD cohort.
