# Supplementary material for: Trans-Ethnic Polygenic Analysis Supports Genetic Overlaps of Lumbar Disc Degeneration With Height, Body Mass Index, and Bone Mineral Density
Source: Front Genet. 2018 Aug 3;9:267. doi: 10.3389/fgene.2018.00267 (PMC6088183; doi:10.3389/fgene.2018.00267)
Supplement: Supplementary file 6 [file Table_6.PDF]

Table S6 Association of the PGS of lumbar spine BMD with disc displacement score after adjusting for different sets of covariates.

Covariates	SNP Predictor	sgn(β)	R^2	*p*-value
Age, Sex	GWAS Hits	+	0.217%	3.10E-02
	PGS P≤1.0E-05	+	0.215%	3.55E-02
Age, Sex, Lumbar Injury	GWAS Hits	+	0.222%	3.16E-02
	PGS P≤1.0E-05	+	0.200%	4.10E-02
Age, Sex, Lumbar Injury, Height	GWAS Hits	+	0.185%	5.13E-02
	PGS P≤1.0E-05	+	0.201%	4.23E-02
Age, Sex, Lumbar Injury, BMI	GWAS Hits	+	0.225%	3.16E-02
	PGS P≤1.0E-05	+	0.225%	3.17E-02
Age, Sex, Lumbar Injury, Height, BMI	GWAS Hits	+	0.198%	4.36E-02
	PGS P≤1.0E-05	+	0.215%	3.55E-02
Age, Sex, Lumbar Injury, Weight	GWAS Hits	+	0.209%	3.85E-02
	PGS P≤1.0E-05	+	0.218%	3.45E-02
