# Supplementary material for: Trans-Ethnic Polygenic Analysis Supports Genetic Overlaps of Lumbar Disc Degeneration With Height, Body Mass Index, and Bone Mineral Density
Source: Front Genet. 2018 Aug 3;9:267. doi: 10.3389/fgene.2018.00267 (PMC6088183; doi:10.3389/fgene.2018.00267)
Supplement: Supplementary file 7 [file Table_7.PDF]

Table S7 Testing the genetic overlap between LDD and serum lipid levels.

Base Phenotype	SNP Sets	N(SNPs)[†]	$R^{2‡}$	Association with disc displacement score[§]			Association with disc degeneration score[§]			Association with LDH requiring surgery		
				sgn(β)	R^2	*p*-value	sgn(β)	R^2	*P*-value	sgn(β)	R^2	*P*-value
LDL-C	Known loci	143	2.13%	-	0.047%	3.22E-01	-	0.022%	4.96E-01	+	0.000%	9.42E-01
	PGS P≤1.0E-05	287	3.15%	-	0.034%	3.95E-01	+	0.058%	2.73E-01	+	0.010%	6.47E-01
HDL-C	Known loci	143	4.73%	+	0.160%	6.80E-02	+	0.018%	5.42E-01	-	0.002%	8.51E-01
TC	Known loci	143	2.82%	-	0.029%	4.37E-01	-	0.021%	5.07E-01	-	0.023%	4.96E-01
TG	Known loci	143	6.96%	-	0.233%	2.73E-02	-	0.043%	3.42E-01	-	0.115%	1.28E-01

Abbreviations: LDL-C, low density lipoprotein cholesterol; HDL-C, high density lipoprotein cholesterol; TC, total cholesterol; TG, triglycerides. For HDL-C, TC and TG, the PGS of known lipid loci showed best prediction performance; so only the results for known loci are shown.

[†] Number of SNPs that passed QC and have minor allele frequency ≥0.01 in the HKDD cohort.

[‡] Variance of lipid phenotypes in HKDD cohort explained by the polygenic score. This quantity is derived from a subset of the HKDD cohort (N=620) that have lipid phenotypes measured by high-throughput proton nuclear magnetic resonance metabolomics approach (Soininen et al. 2009). The four lipid phenotypes were adjusted by age and sex in the HKDD cohort.

[§] Association with disc herniation and degeneration scores is evaluated by linear regression adjusting for age, sex and lumbar spine injury.
