# Supplementary material for: Trans-Ethnic Polygenic Analysis Supports Genetic Overlaps of Lumbar Disc Degeneration With Height, Body Mass Index, and Bone Mineral Density
Source: Front Genet. 2018 Aug 3;9:267. doi: 10.3389/fgene.2018.00267 (PMC6088183; doi:10.3389/fgene.2018.00267)
Supplement: Supplementary file 8 [file Table_8.PDF]

Table S8 Testing the phenotype association between lipid levels and disc herniation and degeneration scores.

Target phenotype	Lipid phenotype	$\hat{\beta}$ (SE)	*p*-value
Disc displacement score	LDL-C	0.028 (0.063)	6.58E-01
	HDL-C	-0.111 (0.084)	1.84E-01
	TC	0.009 (0.033)	7.76E-01
	TG	0.065 (0.065)	3.17E-01
Disc degeneration score	LDL-C	-0.049 (0.177)	7.79E-01
	HDL-C	0.144 (0.235)	5.40E-01
	TC	-0.003 (0.093)	9.76E-01
	TG	-0.227 (0.182)	2.13E-01

Abbreviations: LDL-C, low density lipoprotein cholesterol; HDL-C, high density lipoprotein cholesterol; TC, total cholesterol; TG, triglycerides.

Association between LDD scores and lipid levels is evaluated by multiple linear regression including sex and age as covariates.
