# Supplementary material for: Trans-Ethnic Polygenic Analysis Supports Genetic Overlaps of Lumbar Disc Degeneration With Height, Body Mass Index, and Bone Mineral Density
Source: Front Genet. 2018 Aug 3;9:267. doi: 10.3389/fgene.2018.00267 (PMC6088183; doi:10.3389/fgene.2018.00267)
Supplement: Supplementary file 9 [file Table_9.pdf]

Table S9 Prediction performance of polygenic score using different sets of height and BMI associated SNPs.

Base Phenotype	Known trait-associated SNP set[a]	N(SNPs)[b]	R^2	Association with disc displacement score[c]			Association with disc degeneration score[c]		
				sgn(β)	R^2	*p*-value	sgn(β)	R^2	*p*-value
Height	European GWAS	622	5.76%	+	0.003%	8.04E-01	+	0.135%	9.40E-02
	Asian GWAS	109	3.87%	-	0.013%	5.99E-01	-	0.018%	5.46E-01
	European + Asian GWAS	656	6.99%	-	0.012%	6.24E-01	+	0.001%	8.83E-01
BMI	European GWAS	92	1.24%	+	0.016%	5.64E-01	+	0.053%	2.94E-01
	Asian GWAS	30	1.01%	+	0.122%	1.10E-01	+	0.330%	8.67E-03[d]
	European + Asian GWAS	101	1.63%	+	0.030%	4.26E-01	+	0.112%	1.26E-01

a. Known trait associated SNPs were collected from following references: European GWAS of Height (N=253K; Wood AR et al. 2014. Nat Genet. 46:1173-86), Asian GWAS of Height (Combined Stage I+II N=94K; He M et al. 2015. Hum Mol Genet. 2015. 24:1791-800), European GWAS of BMI (N=234K~322K; Locke AE 2015. Nature. 518:197-206), and Asian GWAS of BMI (Stage I N=87K, Stage II N=5~47K; Wen W et al. 2014. Hum Mol Genet 23:5492-504). Different studies may report different SNPs at the same loci. We applied following procedures to combine results from different studies. When merging between GWAS and the exome-chip study, for each GWAS SNP if there was at least one exome-chip SNP located within 1Mb, it was replaced by the nearest exome-chip SNP. Similarly, when merging European and Asian studies, we replaced each European SNP with the nearest Asian SNP within 1Mb distance.

b. Number of SNPs in the HKDD cohort that passed QC and have minor allele frequency ≥0.01.

c. Association with disc herniation and degeneration scores are evaluated by linear regression adjusting for age, sex and previous spine injury.

d. The association of Asian BMI PGS with disc degeneration score was driven by one SNP rs16858082. Whether or not it represents a pleiotropic locus needs further replication.
