# Supplementary material for: Trans-Ethnic Polygenic Analysis Supports Genetic Overlaps of Lumbar Disc Degeneration With Height, Body Mass Index, and Bone Mineral Density
Source: Front Genet. 2018 Aug 3;9:267. doi: 10.3389/fgene.2018.00267 (PMC6088183; doi:10.3389/fgene.2018.00267)
Supplement: Supplementary file 10 [file Table_10.PDF]

Table S10 Results of the simulation to evaluate the approximation formula for correcting R^2 in extreme selected samples.

h^2	$\bar{M}$	$\hat{R}^2_{pop}$	$\hat{R}^2_{sel}$	$\hat{R}^2_{adj}$ Eq (4)	MSE
Same population, same effect size					
0.025	20	0.025 (0.0007)	0.0653 (0.0017)	0.0247 (0.0005)	0.0164
0.05	53	0.0506 (0.0009)	0.1271 (0.0020)	0.0499 (0.0006)	0.0087
0.075	63	0.0737 (0.0010)	0.1786 (0.0021)	0.0735 (0.0008)	0.0058
0.1	89	0.096 (0.0011)	0.225 (0.0022)	0.0971 (0.0009)	0.0045
Different populations, same effect size					
0.025	24	0.0251 (0.0005)	0.0658 (0.0013)	0.0254 (0.0004)	0.0142
0.05	48	0.0507 (0.0009)	0.1272 (0.0020)	0.0506 (0.0007)	0.0116
0.075	77	0.0733 (0.0009)	0.1777 (0.0019)	0.0735 (0.0008)	0.0051
0.1	94	0.0997 (0.0012)	0.2323 (0.0024)	0.0985 (0.0010)	0.0042
Same population, correlated effect sizes					
0.025	34	0.0133 (0.0006)	0.0355 (0.0015)	0.0134 (0.0005)	0.0414
0.05	47	0.0288 (0.0008)	0.075 (0.0020)	0.0281 (0.0007)	0.015
0.075	102	0.041 (0.0010)	0.1045 (0.0024)	0.0411 (0.0008)	0.0148
0.1	110	0.0533 (0.0011)	0.1331 (0.0026)	0.0535 (0.0010)	0.01
Different population, correlated effect sizes					
0.025	19	0.0129 (0.0005)	0.0344 (0.0013)	0.013 (0.0005)	0.0364
0.05	49	0.024 (0.0007)	0.0629 (0.0018)	0.024 (0.0007)	0.0219
0.075	79	0.0351 (0.0009)	0.0903 (0.0022)	0.0355 (0.0009)	0.0114
0.1	91	0.0467 (0.0010)	0.118 (0.0024)	0.0465 (0.0009)	0.0122

$\bar{M}$: average number of markers simulated. $\hat{R}^2_{pop}$: phenotype variance explained in all testing sample, $\hat{R}^2_{sel}$: phenotype variance explained in the extreme-selected sample, $\hat{R}^2_{adj}$: estimates of $\hat{R}^2_{pop}$ from $\hat{R}^2_{sel}$ based on equation (4). MSE: mean square error of $\hat{R}^2_{adj}$ $:= \overline{\left(\frac{\hat{R}^2_{pop}-\hat{R}^2_{adj}}{\hat{R}^2_{pop}}\right)^2}$
