# Supplementary material for: Trans-Ethnic Polygenic Analysis Supports Genetic Overlaps of Lumbar Disc Degeneration With Height, Body Mass Index, and Bone Mineral Density
Source: Front Genet. 2018 Aug 3;9:267. doi: 10.3389/fgene.2018.00267 (PMC6088183; doi:10.3389/fgene.2018.00267)
Supplement: Supplementary file 11 [file Presentation_1.pdf]

# Trans-ethnic polygenic analysis supports genetic overlaps of lumbar disc degeneration with height, body mass index, and bone mineral density

## Appendix

### Appendix 1: Correction of HKOS's Effects in GEFOS Summary Statistics

The HKOS GWAS was included as part of GEFOS consortium GWAS meta-analysis of BMD (Estrada et al. 2012). To make HKOS GWAS an independent testing data, we corrected the summary statistics of GEFOS by reversing the fixed-effect meta-analysis to subtract the HKOS's contribution.

For each SNP, let $\beta_{\text{meta}}, \text{SE}_{\text{meta}}, \beta_{\text{hk}}, \text{SE}_{\text{hk}}$ be the estimated beta coefficient and standard error resulting from the GEFOS and HKOS analysis respectively. Let $\beta_{\text{eur}}, \text{SE}_{\text{eur}}$ be the beta and standard error for the remaining European populations.

Because the fixed-effect meta-analysis is equivalent to

$$\beta_{\text{meta}} = \frac{\beta_{\text{hk}} \times w_{\text{hk}} + \beta_{\text{eur}} \times w_{\text{eur}}}{w_{\text{hk}} + w_{\text{eur}}}$$

$$\text{SE}_{\text{meta}} = \frac{1}{\sqrt{w_{\text{hk}} + w_{\text{eur}}}}$$

where $w_{\text{hk}} = \frac{1}{\text{SE}_{\text{hk}}^2}, w_{\text{eur}} = \frac{1}{\text{SE}_{\text{eur}}^2}$.

Solving for $\beta_{\text{eur}}, \text{SE}_{\text{eur}}$, we have

$$\begin{aligned} \beta_{\text{eur}} &= \frac{\beta_{\text{meta}} \times (w_{\text{hk}} + w_{\text{eur}}) - \beta_{\text{hk}} \times w_{\text{hk}}}{w_{\text{eur}}} \\ &= \frac{\beta_{meta} \times \frac{1}{\text{SE}_{\text{meta}}^2} - \beta_{hk} \times \frac{1}{\text{SE}_{\text{hk}}^2}}{\frac{1}{\text{SE}_{\text{meta}}^2} - \frac{1}{\text{SE}_{\text{hk}}^2}} \\ \text{SE}_{\text{eur}} &= \frac{1}{\sqrt{w_{\text{eur}}}} = \frac{1}{\sqrt{\frac{1}{\text{SE}_{\text{meta}}^2} - \frac{1}{\text{SE}_{\text{hk}}^2}}} \end{aligned}$$

and corresponding p-value as $p_{\text{eur}} = 1 - 2\Phi\left(\frac{\beta_{\text{eur}}}{\text{SE}_{\text{eur}}}\right)$, where $\Phi$ is the normal cumulative distribution function.

We noted that the GEFOS meta-analysis performed double Genomic Control (GC) correction at both individual cohort and overall meta-analysis results level (Estrada et al. 2012). In practice, we first divided the standard errors of GEFOS summary statistics by the square root of reported second-round inflation factors ($\lambda_{\text{FN-BMD}}$=1.112, $\lambda_{\text{LS-BMD}}$=1.127), and then corrected raw HKOS summary statistics by GC before applying the above correction formula.

In polygenic score (PGS) analysis, sample overlap has large influence when null markers dominate the SNPs included to create PGS. This because at the null, random fluctuation of the test statistics has higher variance in small target sample than the remaining samples. Null markers that happen to show large test statistics in the target sample tend to show the same sign in the overall meta-analysis results. Such systematic errors will be aggregated to inflate $R^2$ at when large number of null markers are included in PGS. Although the above correction procedure should be reasonably accurate to correcting sample overlaps, it may still suffer from numerical errors especially at null markers due to limited precision of shared GWAS summary statistics. In Fig 1C and 1D, $R^2$ in the HKOS sample show slight increase again after SNPs with $P$>0.1 are included. We suspect that this phenomenon does not reflect genetic architecture but is due to aggregated effects of small numerical errors that are direction consistent between the discovery and testing samples. When estimating parameters of BMD genetic architectures, we only used PGS results up to p-value threshold of 0.1.

## Appendix 2: Infer Genetic Architecture from the Results of Polygenic Score Regression

To infer genetic architecture of height and BMI, we fitted the theoretical model of Dudbridge (2013) to the observed PGS results using maximum quasi-likelihood method implemented in AVENGEME (Palla and Dudbridge 2015). Briefly, the model assumes $M$ independent SNPs in the discovery GWAS (sample size $N_1$), a fraction of them ($\pi_0$) are null markers not associated with the phenotype, and the remaining trait-associated SNPs have effect sizes following normal distribution with mean 0 and variance $\sigma_1^2 = \frac{1}{(1-\pi_0)M} h_1^2$ so that the total SNP heritability is $h_1^2$. The genetic covariance of the trait between the discovery and testing sample is $\sigma_{12}$. To fit the observed PGS results, we set $N_1$ to the median sample size across all SNPs and $M$ to the number of clumped SNPs, then $\pi_0$ and $\sigma_{12}$ were estimated by fixing the SNP heritability $h_1^2$ at several plausible values (as we found $h_1^2$ could not be reliably estimated along with $\pi_0$ and $\sigma_{12}$ with the available data).

In practice, GWAS summary statistics were all processed by different GC corrections to minimize the influence of population structures or cryptic relatedness (Winkler et al. 2014). For highly polygenic traits, the standard GC correction is usually over-conservative (Yang et al. 2011) and results in under-estimated SNP heritability (Bulik-Sullivan et al. 2015). It will also cause model mis-specification when fitting the theoretical model to the observed results of PGS regression that make use of public GWAS summary statistics.

The following procedure was applied to approximately address this issue. We first inflated the z-scores of whole-genome summary statistics by different factors and applied LD score regression (Bulik-Sullivan et al. 2015) to estimates SNP heritability. For BMI and Height, we searched through a grid of values from 1 to 1.5 with a step of 0.01. Then we selected the inflation factor $f$ that yielded SNP heritability estimate closest to specified value (0.42 for height and 0.22 for BMI based on previous publications). For BMD, we found that inflating by reported GC correction factors as $f$ could recover reasonable estimates of SNP heritability. Denote $l$ as LD score regression intercept. When fitting the theoretical model to the observed PGS results, we substituted the observed p-value thresholds ($p_o$) with the following corrected ones ($p_c$).

$$p_c = 1 - 2\Phi\left(0 - \frac{f}{l} \cdot \Phi^{-1}\left(\frac{p_o}{2}\right)\right)$$

## Appendix 3: Phenotype Variance Explained in Extreme Selected Samples

The HKOS GWAS sample were selected based on extreme BMD z-scores to improve the power of GWAS. But QTL effect size estimates are upward biased under standard linear regression due to extreme selection. PGS regression in the HKOS sample will also inflate the estimated BMD variance explained ($R^2$). Although there are methods to estimate unbiased QTL effects (Huang and Lin 2007, Kwan, Kung, and Sham 2011), they all require access to the raw genotype and phenotype data. Here we quantify the increase in the $R^2$ by PGS in extreme-selected sample and derive an approximate formula to correct this bias using only summary level data.

We assume there are $m$ independent potential trait associated markers. For locus $i$ ($i = 1, \cdots, m$) reference allele has frequency $p_i$ and true allelic substitution effect $\beta_i$. Genotypes $G_i$ are coded as $X_i - 2p_i$, where $X_i$ is the number of reference alleles, so that $EG_i = 0$. The trait $Y$ is standardized to have mean 0 and variance 1 in the population. $Y$ can be expressed as a linear combination of $m$ genetic effects and an error term that includes environmental and residual genetic effects: $Y = \sum_{i=1}^{m} \beta_i G_i + E$. Genetic effects on $Y$ are first estimated from a discovery GWAS sample of size $n_1$. The estimated allelic substitution effects for locus $i$ is $\hat{\beta}_i$ which has expectation $\beta_i$ and sampling variance $\frac{1}{2p_i(1-p_i))n_1}$ when effect size is small. The estimated effects ($\hat{\beta}$'s) are used to create PGS in an independent sample to be tested for association with trait $Y'$. Assuming testing sample are also genotyped on the same $m$ loci, and $i$-th locus has reference allele frequency $p_i'$. Trait $Y'$ is also standardized and can be related to the genotypes by $Y' = \sum_{i=1}^{m} \beta_i' G_i' + E'$. The PGS in the testing sample is defined as $S = \sum_{i=1}^{m} \hat{\beta}_i G_i'$. The phenotype variance in the testing sample explained by the polygenic score is $R^2_{S,Y'} = \frac{\mathrm{cov}^2(\hat{S},Y')}{\mathrm{var}(\hat{S})\mathrm{var}(Y')}$, where

$$
\begin{aligned}
\mathrm{var}(S) &= \mathrm{var}\left(\sum_{i=1}^{m} \hat{\beta}_i G_i'\right) \\
&= \mathrm{var}\left(\sum_{i=1}^{m} (\hat{\beta}_i - \beta_i) G_i' + \beta_i G_i'\right) \\
&= \sum_{i=1}^{m} \mathrm{var}\left[(\hat{\beta}_i - \beta_i) G_i'\right] + \mathrm{var}\left(\sum_{i=1}^{m} \beta_i G_i'\right) \\
&= \frac{m}{n_1} + \mathrm{var}\left(\sum_{i=1}^{m} \beta_i G_i'\right)
\end{aligned}
$$

$$\begin{aligned}\text{cov}(S,Y') &= \text{cov}\left(\sum_{i=1}^{m}\hat{\beta}_i G_i', \sum_{i=1}^{m}\beta_i' G_i' + E'\right)\\ &= \text{cov}\left(\sum_{i=1}^{m}[(\hat{\beta}_i - \beta_i)G_i' + \beta_i G_i'], \sum_{i=1}^{m}\beta_i' G_i' + E'\right)\\ &= \text{cov}\left(\sum_{i=1}^{m}\beta_i G_i', \sum_{i=1}^{m}\beta_i' G_i'\right)\end{aligned}$$

When the same trait is analyzed in the discovery and testing sample that are from the same homogenous population $\beta_i = \beta_i'$, $G' = \sum_{i=1}^{m}\beta_i G_i'$ is the total genetic value of *m* markers in the testing sample. Since $\text{var}(Y') = 1$, we have

$$R^2_{S,Y'} = \frac{[\text{cov}(S,Y')]^2}{\text{var}(S)} = \frac{[\text{var}(G')]^2}{\text{var}(G') + \frac{m}{n_1}}$$

Similar formulae have been derived by Daetwyler, Villanueva, and Woolliams (2008) and Dudbridge (2013). Here we consider the general case when the discovery and testing sample can be from different populations and make no distributional assumptions about effect sizes.

Now consider the situation when the testing sample is not a random sample of population but only included individuals whose trait value is above or below certain thresholds. We use symbols with ~ to denote values after selection. Tang (2010) showed that when effect sizes are small, genotype mean and variances at each individual locus remain approximately the same after selection $\text{E}(\tilde{G}_i') \approx \text{E}(G_i') = 0, \text{var}(\tilde{G}_i') \approx \text{var}(G_i') = 2p_i(1-p_i)$, whereas the QTL effect and residual trait variance approximately increased by $\tilde{\beta}_i' = f\beta_i'$, $\text{var}(\tilde{E}') = f\text{var}(E')$, where $f = \frac{\text{var}(\tilde{Y}')}{\text{var}(Y')}$. Therefore,

$$\frac{\text{cov}(\tilde{S},\tilde{Y}')}{\text{cov}(S,Y')} \approx \frac{\text{cov}(\sum_{i=1}^{m}\beta_i\tilde{G}_i', \sum_{i=1}^{m}f\beta_i\tilde{G}_i')}{\text{var}(\sum_{i=1}^{m}\beta_i G_i')} = f \qquad (1)$$

Although $\text{var}(\tilde{G}_i') \approx \text{var}(G_i')$ is approximately true at individual loci, when aggregated over *m* markers, the change in variance of $G' = \sum_{i=1}^{m}\beta_i G_i'$ is non-negligible. When effect sizes are small and *m* is large, $G'$ approximately follows a normal distribution. We can write $G'$ as a linear function of $Y'$, $G' = bY' + e$, then $b = \frac{\text{cov}(Y',G')}{\text{var}(Y')} = \text{cov}(\sum_{i=1}^{m}\beta_i G_i', \sum_{i=1}^{m}\beta_i' G_i') \triangleq c\text{var}(G'), \text{var}(e) = \text{var}(G')(1 - c^2\text{var}(G'))$ [Note: when $\text{var}(\beta_i) = \text{var}(\beta_i')$, $c$ can be interpreted as the correlation between $\beta_i$ and $\beta_i'$, and $c = 1$ if $\beta_i = \beta_i'$]. After extreme selection $\tilde{G}' = \sum_{i=1}^{m}\beta_i\tilde{G}_i'$. Since selection on dependent variable $Y'$ does not change the regression coefficient and residual variance, $\tilde{G}' = b\tilde{Y}' + e$. So, we have

$$\mathrm{var}(\tilde{G}') = b^2\mathrm{var}(\tilde{Y}') + \mathrm{var}(e) = \mathrm{var}(G')[1 + (f-1)c^2\mathrm{var}(G')]$$

Therefore,

$$\frac{\mathrm{var}(\tilde{S})}{\mathrm{var}(S)} = \frac{\frac{m}{n_1} + \mathrm{var}\left(\sum_{i=1}^{m}\beta_i\tilde{G}_i'\right)}{\frac{m}{n_1} + \mathrm{var}(\sum_{i=1}^{m}\beta_i G_i')} = \frac{\frac{m}{n_1} + \mathrm{var}(G')[1 + (f-1)c^2\mathrm{var}(G')]}{\frac{m}{n_1} + \mathrm{var}(G')}$$

When $\mathrm{var}(G') \gg \frac{m}{n_1}$, we can have the following approximation

$$\frac{\mathrm{var}(\tilde{S})}{\mathrm{var}(S)} \approx 1 + (f-1)c^2\mathrm{var}(G') \qquad (2)$$

The increase in the phenotype variance explained is therefore:

$$\frac{R^2_{S,\tilde{Y}'}}{R^2_{S,Y'}} = \frac{\frac{[\mathrm{cov}(\tilde{S},\tilde{Y}')]^2}{\mathrm{var}(\tilde{S})\mathrm{var}(\tilde{Y})}}{\frac{[\mathrm{cov}(S,Y')]^2}{\mathrm{var}(S)\mathrm{var}(Y)}} \approx \frac{f}{1 + (f-1)c^2\mathrm{var}(G')} \qquad (3)$$

When the sample size far exceeds the number of markers ($n_1 \gg m$, which is the case for BMD $n_1$≈50K, $m$≈60)

$$R^2_{S,Y'} = \frac{[\mathrm{cov}(\sum_{i=1}^{m}\beta_i G_i', \sum_{i=1}^{m}\beta_i' G_i')]^2}{\frac{m}{n_1} + \mathrm{var}(\sum_{i=1}^{m}\beta_i G_i')} = \frac{c^2[\mathrm{var}(G')]^2}{\frac{m}{n_1} + \mathrm{var}(G')} \approx c^2\mathrm{var}(G')$$

Substituting $c^2\mathrm{var}(G')$ in equation (3) to $R^2_{S,Y'}$ and solving for $R^2_{S,Y'}$, it suggests the following correction formula to estimate $R^2_{S,Y'}$ from $R^2_{S,\tilde{Y}'}$:

$$\hat{R}^2_{S,Y'} \approx \frac{\hat{R}^2_{S,\tilde{Y}'}}{f - (f-1)\hat{R}^2_{S,\tilde{Y}'}} \qquad (4)$$

To validate above formula, we simulated genotype-phenotype data of the discovery and testing samples. When the discovery and testing samples are from the same population, we randomly draw reference allele frequency $p$ from uniform distribution (0.05, 0.95), then sample individual's genotype assuming HWE. When they are from different population, let $F_{ST}$ be the population differentiation, the ancestral allele frequency $p_a$ is uniformly distributed on (0.05,0.95), then subpopulation allele frequencies are sampled from a beta distribution with parameters $\frac{p_a(1-F_{ST})}{F_{ST}}$ and $\frac{(1-p_a)(1-F_{ST})}{F_{ST}}$, so that they have mean $p_a$ and

variance $F_{ST}p_a(1-p_a)$. When we assume the effect size of each marker is the same in the discovery and testing samples, the heritability of each marker $2p(1-p)\beta^2$ is first drawn from a normal distribution of $N(0, h_l^2)$, then substitution effect $\beta$ is derived using allele frequency $p$. When effect sizes in the discovery and testing sample are not assumed to be the same, the heritability of each locus in the discovery and testing sample is jointly sampled from a bivariate normal distribution with zero mean and variance-covariance $\begin{bmatrix} h_l^2 & \rho h_l^2 \\ \rho h_l^2 & h_l^2 \end{bmatrix}$. A total of $M$ QTL are simulated that together contribute to heritability $h^2$ in each sample. The residual phenotype variance has normal distribution of mean 0 and variance $1-h^2$.

Parameters in the simulation are chosen to match the BMD PGS analysis. The sample size is 50,000 GWAS discovery sample and 4000 for testing sample. The heritability of all trait-associated markers $h^2 = 0.025, 0.05, 0.075, 0.1$; the average heritability of each marker $h_l^2 = 0.001$. When they have different effect sizes, the genetic correlation between two samples is $\rho = 0.75$. When simulating different populations, $F_{ST} = 0.1$ that matches the differentiation between European and East Asians. Individuals with extreme phenotype in the testing sample are defined as those whose phenotype below $l = -1.28$ or above $u = 1$. Since phenotype $Y'$ follows standard normal distribution, then the increase in phenotype variance is $f = 1 + \frac{u\phi(u)-l\phi(l)}{1-\Phi(u)+\Phi(l)} - \left(\frac{\phi(u)-\phi(l)}{1-\Phi(u)+\Phi(l)}\right)^2 = 2.739$. We first tested association of each marker in the discovery sample, then used the estimated effect sizes to create PGS in the testing sample and test their association with the phenotypes in selected or all testing sample. Under each parameter set, simulation was repeated 100 times. The results are shown in the Table S10. It demonstrates that the correction formula (4) can accurately recover phenotype variance explained by the PGS in the full testing sample from a phenotype selected subset.

## Appendix 4: Disease Liability Explained by the Polygenic Score

For binary trait, affection status can be modelled using a liability threshold model (Falconer and Mackay 1996), under which all individuals have an underlying normal distributed traits (liability), and all those whose liability exceed a fixed threshold will be affected. Because heritabilities of binary traits are usually reported on the liability scale, the coefficient of determination of PGS on this scale ($R_l^2$) can be compared to heritability and more interpretable than other types of $R^2$.

Assuming disease prevalence in the population is $K$, the proportion of cases in the case-control data is $P$. Define $t = \Phi^{-1}(1-K)$ and $z = \phi(t)$, where $\phi$ and $\Phi$ are standard normal density and cumulative distribution function, respectively. Lee et al. (2012) showed that $R_l^2$ can be converted from $R^2$ on the observed scale by

$$R_l^2 = \frac{R_o^2 C}{1 + R_o^2 \theta C} \tag{5}$$

where $C = \frac{K(1-K)}{z^2}\frac{K(1-K)}{P(1-P)}$ and $\theta = \frac{z}{K}\frac{P-K}{1-K}\left(\frac{z}{K}\frac{P-K}{1-K} - t\right)$; $R_o^2$ is the coefficient of determination using linear regression on the testing data by coding the binary trait as 0-1 response. In power calculation for case-control data, the non-central chi-squared parameter is a function of $R^2$ on the observed scale. $\frac{NR_o^2}{1-R_o^2}$. To related power to $R^2$ on the liability scale, we inversed the above formula to have

$$R_o^2 = \frac{R_l^2}{C - R_l^2 \theta C} \tag{6}$$

When preforming PGS regression using only summary statistics of logistic regression of the testing sample, we first converted $\widehat{\ln\mathrm{OR}}$ and $\mathrm{SE}\left(\widehat{\ln\mathrm{OR}}\right)$ of logistic regression to $\hat{\beta}_{\mathrm{obs}}$ and $\mathrm{SE}\left(\hat{\beta}_{\mathrm{obs}}\right)$ of linear regression, then applied PGS regression under summary statistic mode (Johnson 2012) to calculate $R_o^2$. For small effect sizes, the effect estimate from linear regression can be related to estimated log-odds ratios from logistic regression by the linear approximation (Dudbridge 2013):

$$\hat{\beta}_{\mathrm{obs}} \approx P(1-P)\widehat{\ln\mathrm{OR}}$$

$$\mathrm{SE}\left(\hat{\beta}_{\mathrm{obs}}\right) \approx P(1-P)\mathrm{SE}(\widehat{\ln\mathrm{OR}})$$
