# Supplementary material for: Trans-Ethnic Polygenic Analysis Supports Genetic Overlaps of Lumbar Disc Degeneration With Height, Body Mass Index, and Bone Mineral Density
Source: Front Genet. 2018 Aug 3;9:267. doi: 10.3389/fgene.2018.00267 (PMC6088183; doi:10.3389/fgene.2018.00267)

Figure S1 Prediction performance of PGS on four lipid traits in the HKDD cohort. Lipid traits were measured using magnetic resonance metabolomics technology on a subset of the cohort (*N*=620 genotyped individuals).

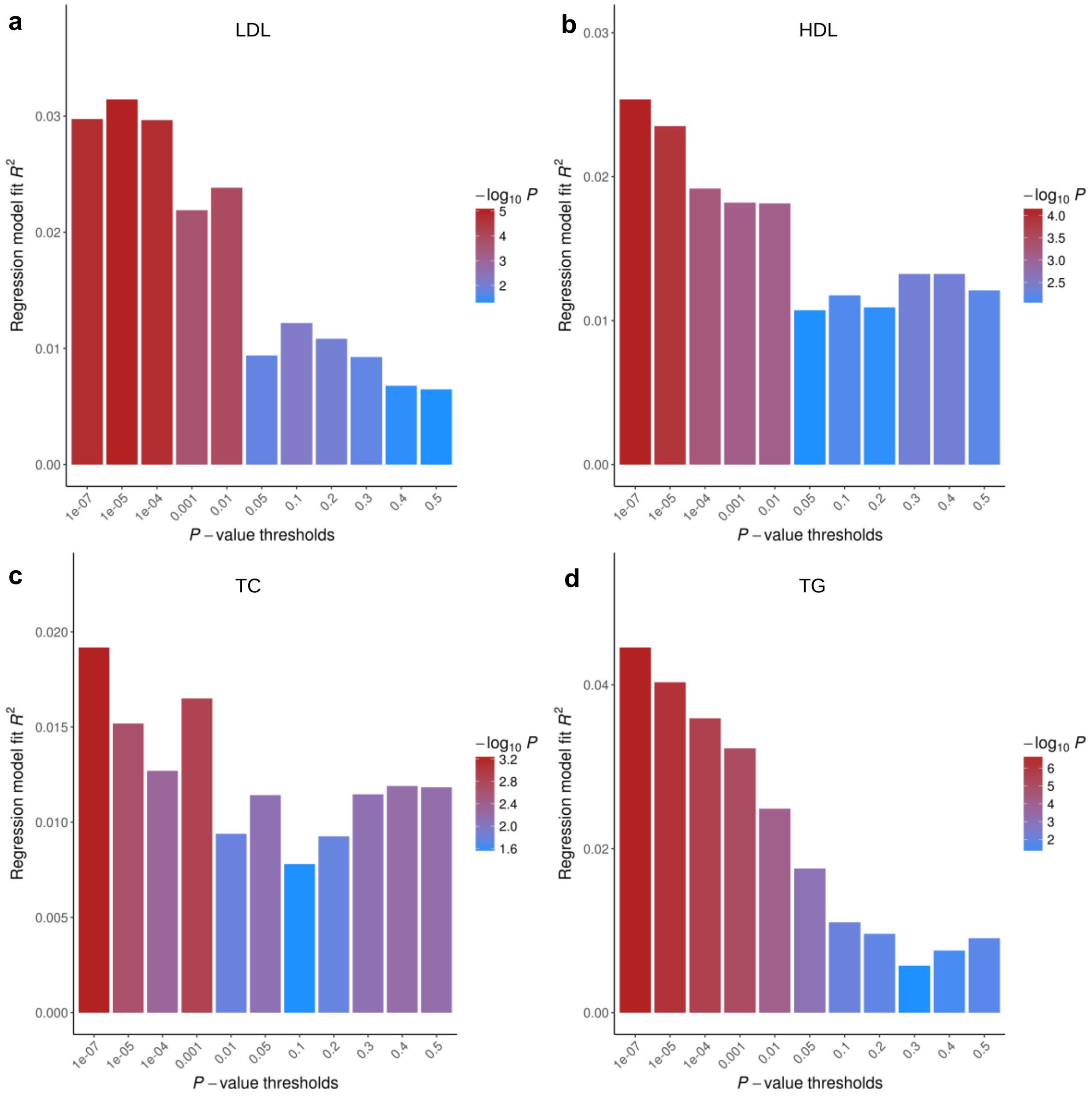

Supplement: Supplementary file 12 [file Image_1.PDF]
