# Supplementary material for: Trans-Ethnic Polygenic Analysis Supports Genetic Overlaps of Lumbar Disc Degeneration With Height, Body Mass Index, and Bone Mineral Density
Source: Front Genet. 2018 Aug 3;9:267. doi: 10.3389/fgene.2018.00267 (PMC6088183; doi:10.3389/fgene.2018.00267)
Supplement: Supplementary file 13 [file Image_2.pdf]

Figure S2 Comparing the prediction performance of PGS in Chinese population using discovery GWAS of European and East Asian under different parameter sets. The figure is the same as Figure 3, but at different values of presumed SNP heritabilities for height and BMI.

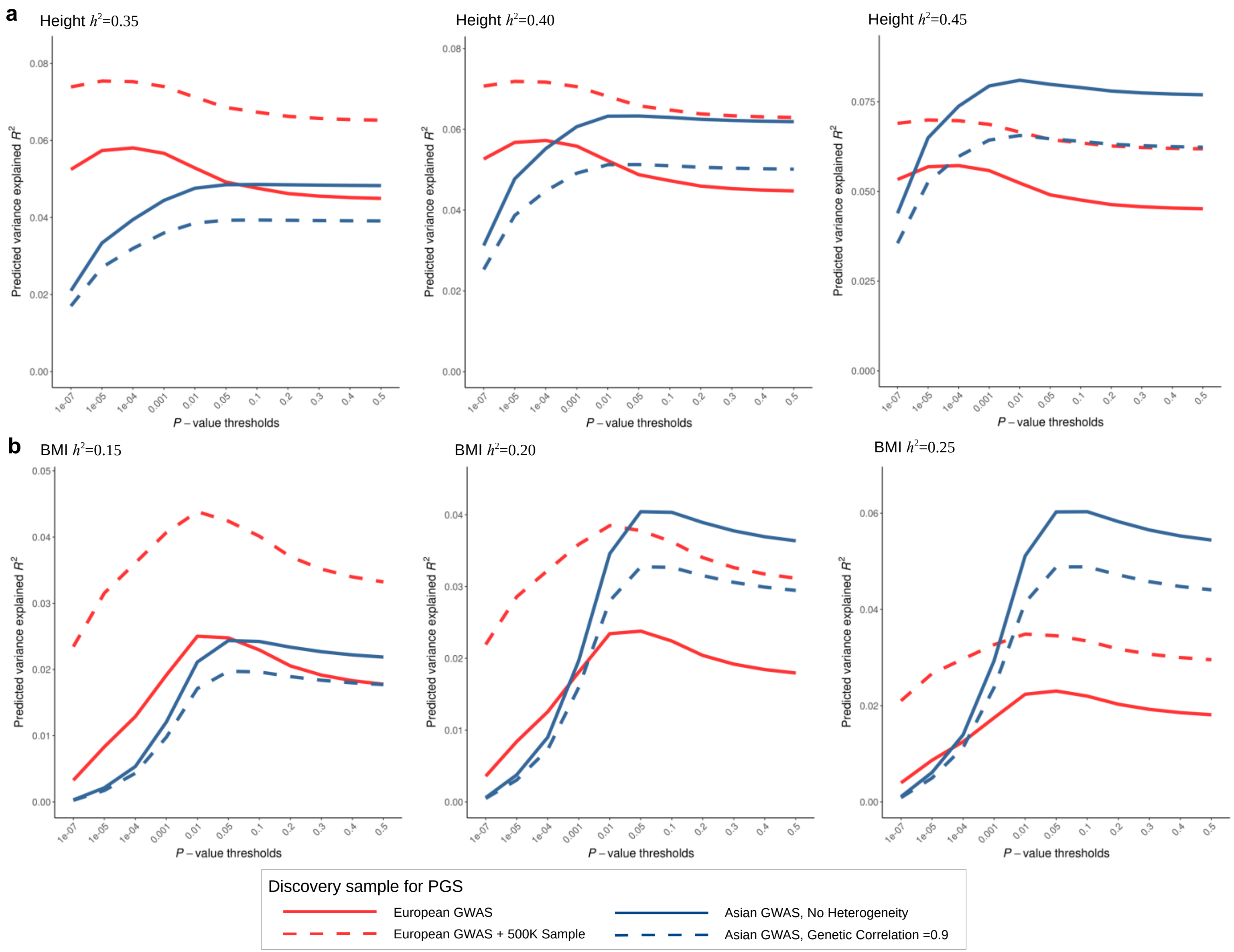
a
Height h^2=0.35
Height h^2=0.40
Height h^2=0.45
b
BMI h^2=0.15
BMI h^2=0.20
BMI h^2=0.25
Predicted variance explained R^2
P – value thresholds
1e-07
1e-05
1e-04
0.001
0.01
0.05
0.1
0.2
0.3
0.4
0.5
Discovery sample for PGS
European GWAS
European GWAS + 500K Sample
Asian GWAS, No Heterogeneity
Asian GWAS, Genetic Correlation =0.9
